# Correlation of SARS-CoV-2 Subgenomic RNA with Antigen Detection in Nasal Midturbinate Swab Specimens

**DOI:** 10.3201/eid2711.211135

**Published:** 2021-11

**Authors:** Katherine Immergluck, Mark D. Gonzalez, Jennifer K. Frediani, Joshua M. Levy, Janet Figueroa, Anna Wood, Beverly B. Rogers, Jared O’Neal, Roger Elias-Marcellin, Allie Suessmith, Julie Sullivan, Raymond F. Schinazi, Ahmed Babiker, Anne Piantadosi, Miriam B. Vos, Greg S. Martin, Wilbur A. Lam, Jesse J. Waggoner

**Affiliations:** Emory University, Atlanta, Georgia, USA (K. Immergluck, M.D. Gonzalez, J.K. Frediani, J.M. Levy, J. Figueroa, A. Wood, B.B. Rogers, J. O’Neal, R. Elias-Marcellin, A. Suessmith, J. Sullivan, R.F. Schinazi, A. Babiker, A. Piantadosi, M.B. Vos, G.S. Martin, W.A. Lam, J.J. Waggoner);; The Atlanta Center for Microsystems-Engineered Point-of-Care Technologies, Atlanta (K. Immergluck, M.D. Gonzalez, J.K. Frediani, J.M. Levy, J. Figueroa, A. Wood, B.B. Rogers, J. O’Neal, A. Suessmith, J. Sullivan, R.F. Schinazi, M.B. Vos, G.S. Martin, W.A. Lam, J.J. Waggoner);; Children’s Healthcare of Atlanta, Atlanta (M.D. Gonzalez, M.B. Vos)

**Keywords:** severe acute respiratory syndrome coronavirus 2, SARS-CoV-2, coronavirus, viruses, coronavirus disease, COVID-19, respiratory infections, RNA, subgenomic RNA, nucleocapsid, antigen detection, nasal midturbinate, nasopharyngeal, swab specimens, zoonoses

## Abstract

Among symptomatic outpatients, subgenomic RNA of severe acute respiratory syndrome coronavirus 2 in nasal midturbinate swab specimens was concordant with antigen detection but remained detectable in 13 (82.1%) of 16 nasopharyngeal swab specimens from antigen-negative persons. Subgenomic RNA in midturbinate swab specimens might be useful for routine diagnostics to identify active virus replication.

Accurate detection of severe acute respiratory syndrome coronavirus 2 (SARS-CoV-2) infection is critical for patient management and infection control (*1*). Molecular diagnostics are highly sensitive in the acute phase of coronavirus diseases (COVID-19), but viral RNA remains detectable long after replicating virus can be isolated from respiratory samples (*1*–*5*). Antigen diagnostics, though often less sensitive, are touted as providing accurate detection during peak infectivity, thereby identifying persons most likely to transmit SARS-CoV-2 (*6*,*7*).

Prolonged SARS-CoV-2 RNA detection has led to evaluation of molecular assays to detect subgenomic RNA (sgRNA) or negative-strand RNA, which are produced during active viral replication (*2*–*5*,*8*–*10*). sgRNA detection has predominantly been studied in hospitalized adults who have COVID-19 (*2*,*3*,*5*,*8*,*9*); published reports have not compared sgRNA and antigen detection, which should be highly correlated. We compared real-time reverse transcription PCR (rRT-PCR) detection of nucleocapsid sgRNA, the most abundant sgRNA in SARS-CoV-2‒infected cells (*2*), with nucleocapsid antigen detection among symptomatic outpatients who had SARS-CoV-2 infections.

## The Study

We obtained 88 nasal midturbinate and 39 nasopharyngeal swab specimens (PurFlock Ultra Flocked Swabs; Puritan Medical Products, https://www.puritanmedproducts.com) from 127 persons who came to COVID-19 testing centers affiliated with Emory University and Children’s Healthcare of Atlanta (Atlanta, GA, USA) during January 2021. Inclusion criteria were a symptomatic respiratory illness for <7 days and a positive, routine-care SARS-CoV-2 molecular test (nasopharyngeal swab specimen). The study was approved by the Emory University Institutional Review Board and Children’s Healthcare of Atlanta.

We extracted total nucleic acids from 500 μL of sample and eluted them into a volume of 50 μL by using an EMAG Instrument (bioMérieux, https://www.biomerieux.com). We tested eluates side-by-side in rRT-PCRs for sgRNA and total SARS-CoV-2 RNA (genomic plus sgRNA). For sgRNA, we combined a forward primer in the leader sequence (5′-CGATCTCTTGTAGATCTGTTCTC-3′) with the nucleocapsid 2 (N2) target reverse primer and probe (*11*).

We performed the sgRNA assay in 20-μL reactions using the Luna Probe One-Step RT-qPCR Kit (New England Biolabs, https://www.neb.com) with 500 nmol/L of each primer, 250 nmol/L of probe, and 5 μL of eluate by using the following conditions: 55°C for 15 min, 95°C for 2 min, and 45 cycles of 95°C for 15 s and 60°C for 60 s. We detected total SARS-CoV-2 RNA by using a duplex N2-RNase P rRT-PCR performed as described (*12*). We obtained an anterior nares swab specimen for nucleocapsid antigen detection with the Abbott BinaxNOW COVID-19 Ag Card (swabs supplied with the BinaxNOW kit; Abbott Laboratories, https://www.abbott.com) performed per the package insert.

The first 73 participants had a midturbinate swab specimen available for molecular testing (evaluation group) and have been described (*13*). The subsequent 54 participants had dedicated midturbinate (n = 15) or residual nasopharyngeal (n = 39) swab specimens for molecular testing and available antigen test results (antigen testing group) (Appendix Figure 1).

We estimated nucleocapsid sgRNA as a percentage of total RNA by calculating copies per microliter of sgRNA and total RNA for each sample based on a standard curve for each target and then calculating the percentage of sgRNA. We used unpaired t-tests to compare continuous variables and the Fisher exact test for testing categorical variables. We performed simple linear regression to compare cycle threshold (C_t_ ) values for sgRNA and total RNA. We conducted analyses by using GraphPad version 9.02 (https://www.graphpad.com) and SAS version 9.4 (https://support.sas.com).

**Figure 1:**
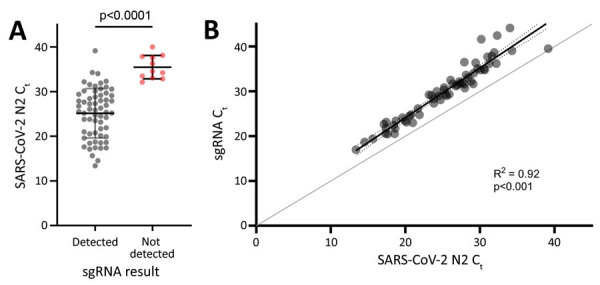
Correlation of sgRNA levels with total SARS-CoV-2 RNA in samples from study participants in Atlanta, Georgia, USA. A) N2 C_t_ values for samples in which sgRNA was detectable (gray dots) or not detectable (red dots). Horizontal bars indicate means, and error bars indicate SDs. B) sgRNA C_t_ values versus corresponding C_t_ values for the N2 target. Results of simple linear regression (black line) and error bars (dotted lines) are shown. Line of identity (gray line) is shown for reference. C_t_, cycle threshold; N2, nucleocapsid 2; SARS-CoV-2, severe acute respiratory syndrome coronavirus 2; sgRNA, subgenomic RNA.

The evaluation group included midturbinate swab specimens from 36 adults and 37 children. All samples (73/73) were positive for SARS-CoV-2 by rRT-PCR. Samples with detectable sgRNA (62/73, 84.9%) had significantly lower C_t_ values, indicative of higher viral loads, than samples without detectable sgRNA (mean C_t_ 25.1, SD 5.5, vs. mean C_t_ 35.5, SD 2.6; p<0.0001) (Figure 1, panel A). sgRNA was detectable in all samples (49/49) that had N2 C_t_ values <30 compared with 13 (54.2%) of 24 samples that had C_t_ values >30 (p<0.0001). Although sgRNA rRT-PCR amplification efficiency was slightly lower than that for the N2 assay, there was a strong linear correlation between sgRNA and N2 C_t_ values (Figure 1, panel B), and the assay provided linear sgRNA detection across the range of N2 C_t_ values observed in this study (Appendix Figure 2). sgRNA C_t_ values were a mean of 4.8 (SD 1.8) cycles higher than corresponding N2 C_t_ values, and nucleocapsid gene sgRNA accounted for a mean of 1.4% (SD 1.1%) of SARS-CoV-2 RNA. Samples from children had higher viral loads than samples from adults, although the relative amount of sgRNA did not differ (Appendix Figure 3).

**Figure 2:**
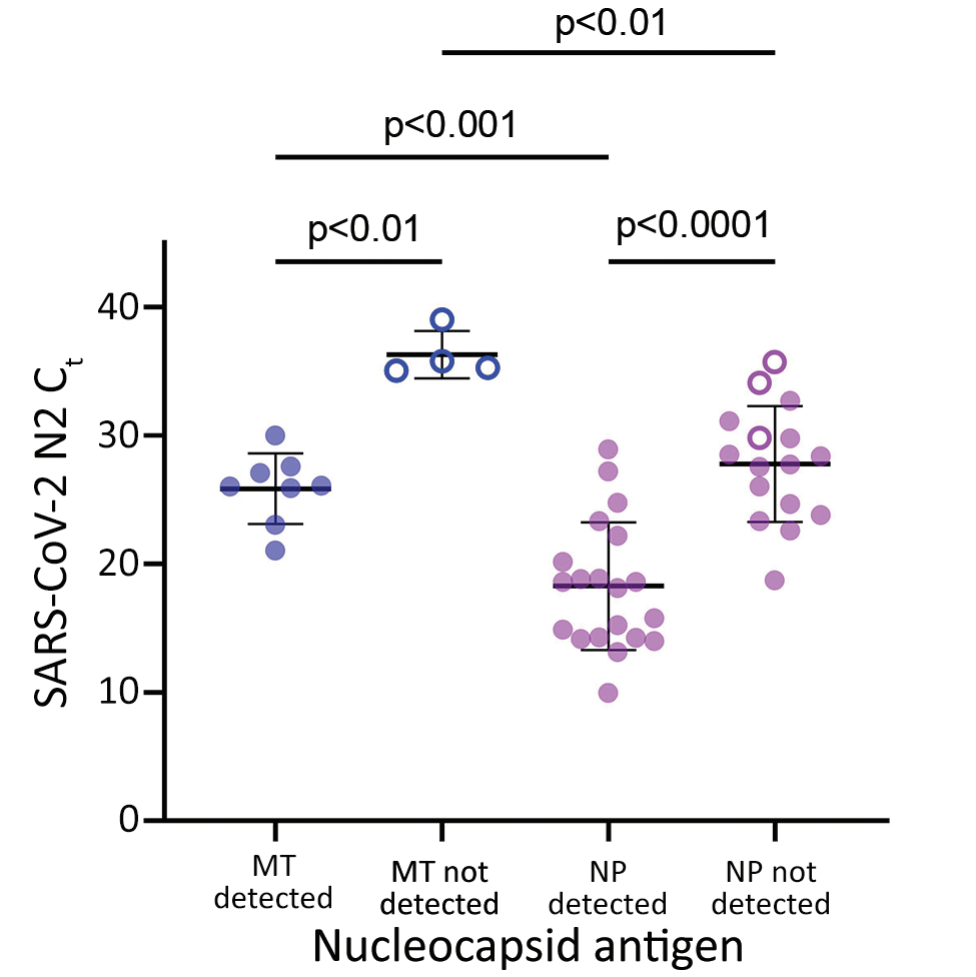
Concordance of SARS-CoV-2 sgRNA with nucleocapsid antigen detection in MT swab specimens, but not NP swab specimens, from study participants in Atlanta, Georgia, USA. sgRNA remains detectable in NP swab specimens for persons who showed negative results for nucleocapsid antigen. Symbols represent MT (blue) and NP (purple) swab specimens for persons with (filled circles) and without (open circles) detectable sgRNA. Horizontal bars indicate means, and error bars indicate SDs. C_t_, cycle threshold; MT, nasal midturbinate; NP, nasopharyngeal; N2, nucleocapsid 2; SARS-CoV-2, severe acute respiratory syndrome coronavirus 2; sgRNA, subgenomic RNA.

We complied characteristics of participants in the antigen-testing group who had midturbinate (n = 15) swab specimens (Table 1) and nasopharyngeal (n = 39) swab specimens (Table 2). All midturbinate swab specimens from participants who had detectable antigen (n = 8) were also positive for sgRNA, whereas 0/4 samples from antigen-negative persons were positive (κ 1.0). Samples that had detectable sgRNA had significantly lower C_t_ values (mean 25.8, SD 2.7) than samples that did not have detectable sgRNA (mean 36.3, SD 1.8; p = 0.002) (Figure 2).

**Table 1:** Demographic and clinical variables of study participants who had MT swab specimens in antigen-testing group analyzed for SARS-CoV-2 subgenomic RNA, Atlanta, Georgia, USA*

Variable	Overall, n = 15	Antigen positive, n = 8	Antigen negative, n = 7	p value
Mean age, y (SD)	54.35 (14.49)	53.98 (16.12)	54.78 (13.65)	0.921
Female sex	9 (60.00)	5 (62.50)	4 (57.14)	1.000
Mean days after symptom onset (SD)†	4.14 (2.44)	3.88 (2.23)	4.50 (2.88)	0.655
MT swab specimen, rRT-PCR positive	12 (80.0)	8 (100.0)	4 (57.1)	0.077
Race‡				
White	2 (14.3)	0	3 (33.3)	0.026
Black/African American	11 (78.6)	8 (100.0)	3 (50.0)	NA
Asian	11 (78.6)	8 (100.0)	3 (50.0)	NA

*Values are no. (%) unless indicated otherwise. MT, nasal midturbinate; NA, not available; rRT-PCR, real-time reverse transcription PCR; SARS-CoV-2, severe acute respiratory syndrome coronavirus 2. †Day sample was collected. ‡One participant did not identify race; none identified as Hispanic.

**Table 2:** Correlation of SARS-CoV-2 subgenomic RNA with nucleocapsid detection in NP specimens from study participants analyzed for SARS-CoV-2 subgenomic RNA, Atlanta, Georgia, USA *

Variable	Overall, n = 39	Antigen positive, n = 20	Antigen negative, n = 19	p value
Mean age, y (SD)	8.6 (5.8)	9.8 (5.6)	7.4 (5.8)	0.148
Female sex	16 (41.0)	8 (40.0)	8 (42.1)	0.894
Mean days after symptom onset (SD)†	3.7 (2.2)	3.0 (1.4)	4.5 (2.7)	0.227
Repeat NP swab specimen, rRT-PCR positive	36 (92.3)	20 (100.0)	16 (84.2)	0.106
Race				
White	26 (66.67)	13 (65.0)	13 (68.42)	0.077
Black/African American	6 (15.38)	3 (15.0)	3 (15.79)	NA
Asian	4 (10.26)	4 (20.0)	0	NA
Biracial	3 (7.69)	0	3 (15.79)	NA
Hispanic ethnicity	19 (48.72)	6 (30.0)	13 (68.42)	0.016

*Values are no. (%) unless indicated otherwise. NA, not available; NP, nasopharyngeal; rRT-PCR, real-time reverse transcription PCR; SARS-CoV-2, severe acute respiratory syndrome coronavirus 2. †Day sample was collected. One participant was asymptomatic (did not report symptoms in the past 14 days).

All (20/20) nasopharyngeal swab specimens from antigen-positive participants were positive for sgRNA. N2 C_t_ values were significantly lower among antigen-positive participants (mean 18.2, SD 5.0) than antigen-negative participants (mean 27.8, SD 4.5; p<0.0001) (Figure 2). sgRNA was detectable in 13 (81.2%) of 16 nasopharyngeal swab specimens from antigen-negative persons. Days after symptom onset (when the sample was collected) did not differ significantly between antigen-positive and sgRNA-positive/antigen-negative participants (mean 3.4, SD 1.9 days, vs. mean 3.8, SD 2.4 days; p = 0.6). Nucleocapsid gene sgRNA accounted for a smaller percentage of total SARS-CoV-2 RNA in antigen-negative participants (mean 0.6%, SD 0.4%) vs. antigen-positive participants (mean 1.0%, SD 0.5%; p = 0.012) (Appendix Figure 4). Compared with midturbinate swab specimens, nasopharyngeal swab specimens had lower C_t_ values for RNase P (Appendix Figure 5).

## Conclusions

SARS-CoV-2 sgRNA was detected in all samples from antigen-positive participants (28/28 total), consistent with identification of active viral replication and potential shedding (*4*,*5*,*8*). However, among antigen-negative participants, sgRNA detection varied between SARS-CoV-2 RNA-positive midturbinate (0/4) and nasopharyngeal (13/16) swab specimens. Although nasopharyngeal swab specimens are expected to have higher viral loads (*14*), this difference did not appear to be the sole explanation. sgRNA represented a smaller proportion of total SARS-CoV-2 RNA in discordant nasopharyngeal swab specimens, and overall, nasopharyngeal swab specimens had higher amounts of human cellular material (lower RNase P C_t_ values) than midturbinate swab specimens. Therefore, discordant sgRNA and antigen results in nasopharyngeal swab specimens probably resulted from persistent detection of waning SARS-CoV-2 infections with low levels of detectable sgRNA, which is only found in infected cells but insufficient viral replication to yield detectable nucleocapsid antigen in the anterior nares.

Nucleocapsid antigen was detected by using the widely available BinaxNOW COVID-19 Ag Card. This card demonstrates similar performance to other rapid antigen tests, which commonly detect nucleocapsid protein, and maintains analytical sensitivity against SARS-CoV-2 variants (*7*). Therefore, it provided a useful and relevant comparator for sgRNA detection.

Limitations of our study include a relatively small number of midturbinate swab specimens tested in the antigen-testing group, which was affected by the need for multiple swab specimens at a single time point. The race/ethnicity makeup of groups that had midturbinate and nasopharyngeal swab specimens differed (Tables 1, 2), although this limitation is not expected to have affected our findings (*15*).

In conclusion, sgRNA detection in midturbinate swab specimens correlates with nucleocapsid antigen and could be implemented as a molecular test to evaluate infectivity. Given the strong correlation between sgRNA, nucleocapsid antigen, and total SARS-CoV-2 RNA, these data also support use of antigen testing or establishment of rRT-PCR C_t_ values as markers of active replication.

Appendix Additional information on correlation of SARS-CoV-2 subgenomic RNA with antigen detection in swab specimens of nasal midturbinate.
