# Supplementary material for: Correlation of SARS-CoV-2 Subgenomic RNA with Antigen Detection in Nasal Midturbinate Swab Specimens
Source: Emerg Infect Dis. 2021 Nov;27(11):2887–91. doi: 10.3201/eid2711.211135 (PMC8544990; doi:10.3201/eid2711.211135)
Supplement: Appendix — Additional information on correlation of SARS-CoV-2 subgenomic RNA with antigen detection in swab specimens of nasal midturbinate. [file 21-1135-Techapp-s1.pdf]

Article DOI: https://doi.org/10.3201/eid2711.211135

Correlation of SARS-CoV-2 Subgenomic RNA with Antigen Detection in Nasal Midturbinate Swab Specimens

Appendix

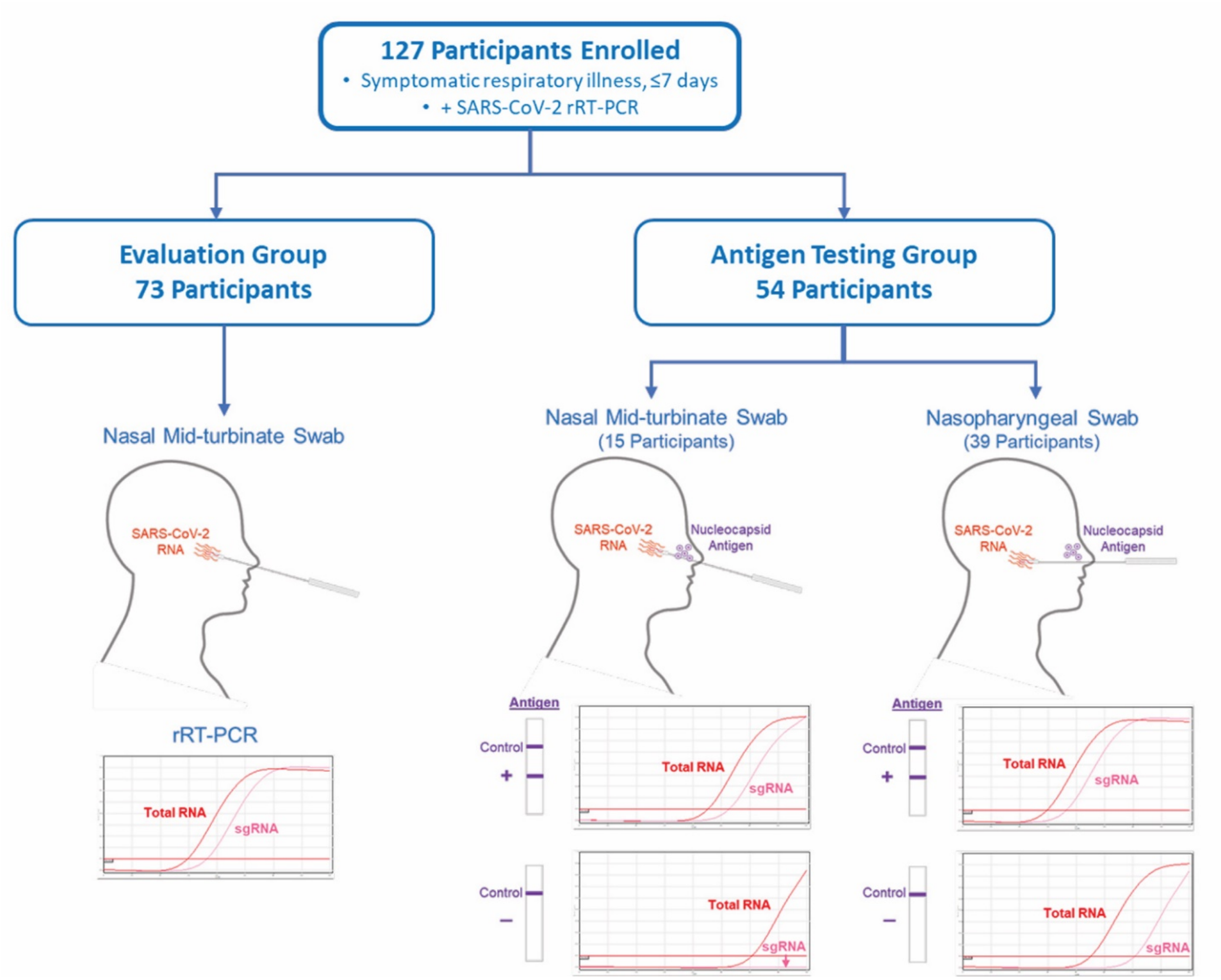

Appendix Figure 1. Study flowchart for 127 enrolled participants. Those in the evaluation group had a nasal midturbinate swab tested for total SARS-CoV-2 RNA and N gene containing sgRNA. Participants in the antigen-testing group had nucleocapsid antigen detection in the anterior nares compared with SARS-CoV-2 total RNA and sgRNA detected in nasal midturbinate or nasopharyngeal swab specimens. rRT-PCR, real-time reverse transcription PCR; SARS-CoV-2, severe acute respiratory syndrome coronavirus 2; sgRNA, subgenomic RNA.

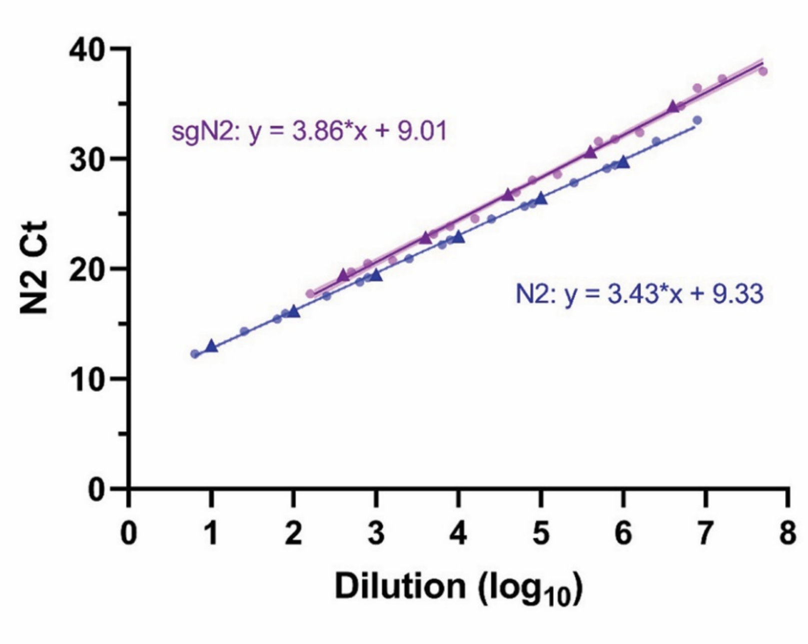

Appendix Figure 2. Linear regression of rRT-PCR results for sgRNA (sgN2, purples line) and total severe acute respiratory syndrome coronavirus 2 (SARS-CoV-2 RNA) (N2, blue line) using a 10-fold dilution series of 4 nasal midturbinate samples from the evaluation group. A standard curve was generated by using sgRNA and total RNA C_t values from a single sample (purple and blue triangles, respectively), accounting for sgRNA abundance relative to total RNA when plotting the first dilution. Series from the remaining samples were normalized to these standard curves and all data was incorporated in a linear regression. Plot displays the linear regression line and 95% CI (shaded region). Efficiency of rRT-PCRs based on this regression are 95.6% for N2 and 81.3% for sgN2. C_t, cycle threshold; rRT-PCR, real-time reverse transcription PCR; sgRNA, subgenomic RNA.

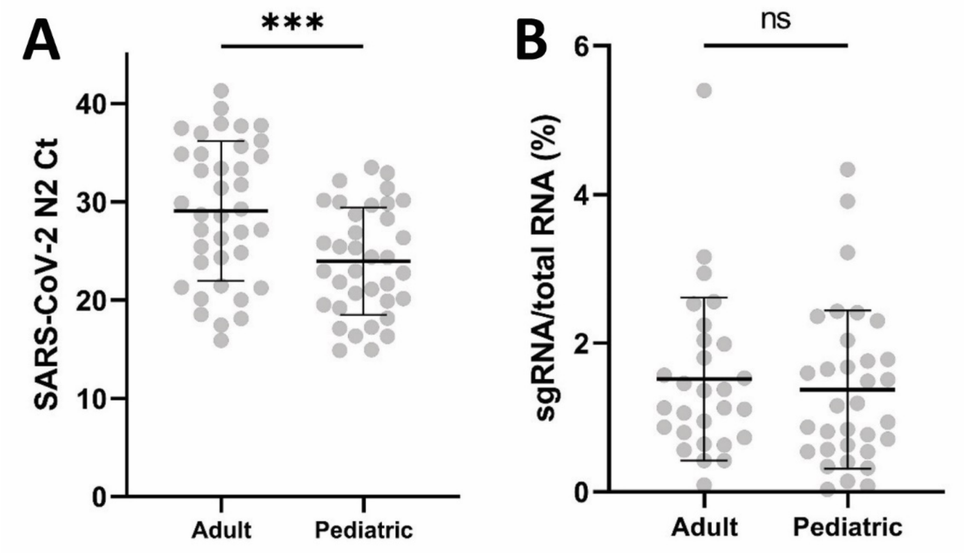

Appendix Figure 3. Distribution of SARS-CoV-2 rRT-PCR, real-time reverse transcription PCR (rRT-PCR) C_t, values (N2 target) in adult and pediatric nasal midturbinate swab specimens from A) the evaluation group and B) comparison of the percentage of total SARS-CoV-2 RNA accounted for by sgRNA. Horizontal bars indicated means, and error bars indicate SDs. C_t, cycle threshold; ns, not significant; SARS-CoV-2, severe acute respiratory syndrome coronavirus 2; sgRNA, subgenomic RNA. ***p<0.001.

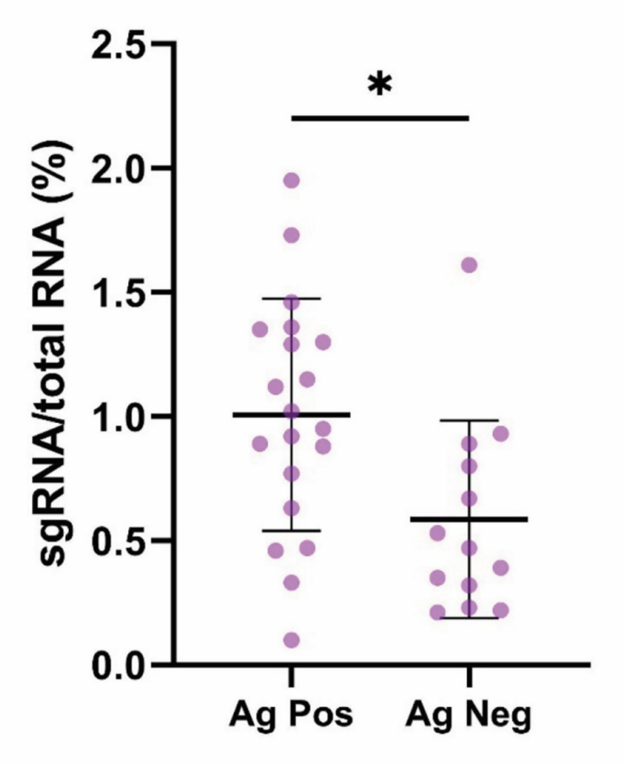

Appendix Figure 4. Percentage of severe acute respiratory syndrome coronavirus 2 (SARS-CoV-2) total RNA accounted for by sgRNA in nasopharyngeal swab specimens from participants who were positive or negative for nucleocapsid antigen. Horizontal bars indicate means, and error bars indicate SDs. Ag, antigen; Neg, negative; Pos, positive; sgRNA, subgenomic RNA. *p<0.05.

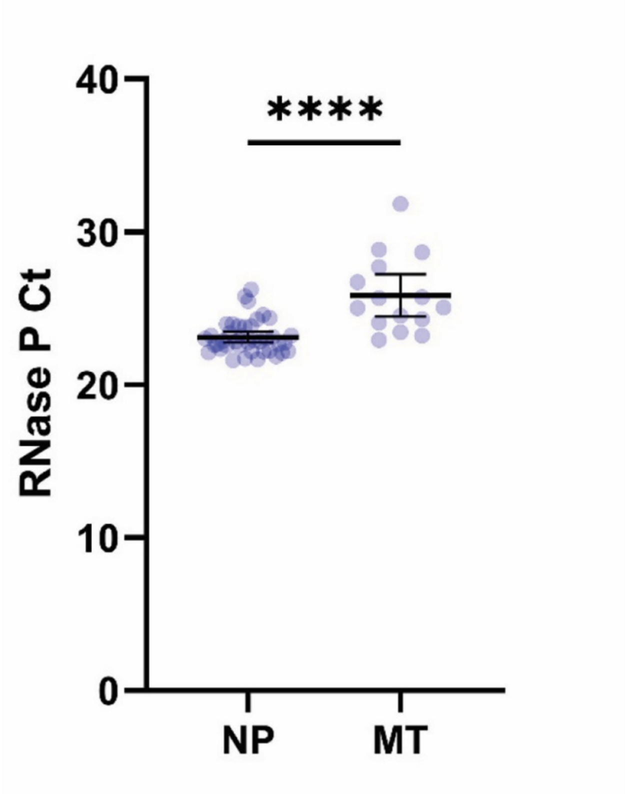

Appendix Figure 5. RNase P C_t values for NP and MT swab specimens in the antigen-testing group. NP swab specimens had lower C_t values (mean 23.1, SD 1.1) than s MT swab specimens (mean 25.85, SD 2.5). Horizontal bars indicate means, and error bars indicated SDs. C_t, cycle threshold; MT, nasal midturbinate; NP, nasopharyngeal. ****p<0.0001.
